# Evolutionary Genomics of Human Gut Bacteria: Ecological Plasticity Across the Mutualism–Pathogenicity Spectrum

**DOI:** 10.3390/ijms27115009

**Published:** 2026-06-01

**Authors:** Yasmin N. Ramadan, Salwa Q. Bukhari, Zinab Alatawi, Ghaleb Oriquat, Noura H. Abd Ellah, Eltayib Hassan Ahmad Mohamedosman, Rehab Ahmed, Helal F. Hetta

**Affiliations:** 1 Department of Microbiology and Immunology, Faculty of Pharmacy, Assiut University, Assiut 71515, Egypt; 2 Department of Radiology, Faculty of Medicine, University of Tabuk, Tabuk 47512, Saudi Arabia; s.bukhari@ut.edu.sa; 3 Department of Family and Community Medicine, Faculty of Medicine, University of Tabuk, Tabuk 47512, Saudi Arabia; zalatawi@ut.edu.sa; 4 Faculty of Allied Medical Sciences, Hourani Center for Applied Scientific Research, Al-Ahliyya Amman University, Amman 19328, Jordan; goreqat@ammanu.edu.jo; 5 Department of Pharmaceutics, Faculty of Pharmacy, Assiut University, Assiut 71515, Egypt; nora.1512@aun.edu.eg; 6 Department of Medical Laboratory Technology, Faculty of Applied Medical Science, University of Tabuk, Tabuk 71491, Saudi Arabia; 7 Division of Microbiology, Immunology and Biotechnology, Department of Natural Products and Alternative Medicine, Faculty of Pharmacy, University of Tabuk, Tabuk 71491, Saudi Arabiahhussen@ut.edu.sa (H.F.H.)

**Keywords:** human gut microbiome, evolutionary genomics, mutualism–pathogenicity spectrum, pathobionts, endosymbionts

## Abstract

The human gut microbiome comprises a diverse community of bacteria whose interactions with the host range from beneficial mutualism to opportunistic pathogenicity. These interactions are shaped by genomic plasticity and ecological pressures that influence whether microbes support host health, remain conditionally harmless, or contribute to disease. Understanding the mechanisms underlying these shifts is essential for clarifying the balance between cooperation and pathogenicity within the gut ecosystem. This review explores the genomic and evolutionary mechanisms that shape microbial adaptation across the mutualism–pathogenicity spectrum in the human gut. Key processes, including horizontal gene transfer (HGT), host-mediated selection, and niche specialization, enable microbes to acquire, regulate, or retain traits that influence colonization, metabolic function, and virulence. These adaptive mechanisms allow gut bacteria to respond dynamically to ecological pressures such as inflammation, antibiotic exposure, and dietary change, resulting in context-dependent microbial behaviors. The review also considers how concepts from insect endosymbiosis may provide insight into gut microbial adaptation. While both systems exhibit host specialization, major differences in transmission mode, ecological flexibility, and genome evolution limit direct comparisons. Rather than following a fixed progression toward parasitism, gut microbes exhibit flexible adaptive strategies shaped by host and environmental conditions. By integrating ecological and evolutionary perspectives, this review presents a balanced framework for understanding how genomic adaptation influences microbial behavior in the gut. This perspective improves our understanding of dysbiosis and microbial pathogenesis and may support the development of microbiome-informed therapeutic strategies for maintaining host health.

## 1. Introduction

The human gastrointestinal tract harbors a dense and diverse microbial community that contributes fundamentally to digestion, nutrient metabolism, immune development, and protection against invading pathogens. These microorganisms are integral to host physiology, yet their interactions with the host are not uniformly beneficial. Depending on ecological context, microbial genetic traits, and host conditions, gut bacteria may promote health, remain functionally neutral, or contribute to disease. This dynamic range of interactions reflects a mutualism–pathogenicity spectrum, in which microbial behavior is shaped by both ecological pressures and evolutionary adaptation [1,2,3].

Insights from classical symbiosis research offer useful perspectives for understanding these host–microbe relationships. In insect systems, obligate endosymbionts such as *Buchnera aphidicola* have evolved highly specialized associations characterized by metabolic interdependence, genome reduction, and long-term host adaptation [4,5,6]. Although human gut microbes differ fundamentally from these intracellular symbionts in transmission mode, ecological flexibility, and genomic diversity, they exhibit comparable adaptive strategies, including niche specialization and host-associated functional adaptation [7,8]. For example, *Bacteroides* species encode extensive polysaccharide utilization loci that support efficient metabolism of host-derived and dietary glycans, while *Akkermansia muciniphila* is highly specialized for mucin degradation within the intestinal mucus layer [9,10,11,12,13]. These examples illustrate how long-term residence in the gut can favor the evolution of traits that enhance persistence within host-associated niches.

At the same time, not all host-adapted microbes remain beneficial. Certain commensal organisms act as pathobionts, remaining harmless under homeostatic conditions but becoming pathogenic when ecological balance is disrupted by inflammation, immune dysfunction, or antibiotic exposure [14,15]. This functional shift is often linked to genomic plasticity, including the acquisition or regulation of virulence determinants, pathogenicity islands, and stress-response systems that enhance colonization and persistence under adverse conditions [16]. Opportunistic pathogens such as *Enterococcus faecalis*, pathogenic *Escherichia coli*, and *Klebsiella pneumoniae* demonstrate how adaptive genomic traits can increase pathogenic potential while preserving the ability to colonize the gut environment [17,18,19,20,21,22].

Understanding how microbial lineages balance cooperative and pathogenic traits is essential for clarifying the evolutionary dynamics of host-associated bacteria. In this review, we examine the genomic and ecological mechanisms that shape microbial adaptation across the mutualism–pathogenicity spectrum in the human gut, with emphasis on horizontal gene transfer (HGT), host-mediated selection, regulatory adaptation, and niche specialization. Rather than acting independently, these mechanisms interact dynamically to influence microbial persistence, ecological behavior, and context-dependent transitions between mutualistic and pathogenic states. We also evaluate how concepts from insect endosymbiosis can inform our understanding of gut microbial adaptation while recognizing the important biological differences between these systems. By integrating ecological and evolutionary perspectives, this review aims to provide a unified framework for understanding how genomic plasticity and environmental pressures collectively shape microbial behavior and its consequences for human health.

## 2. Literature Search Strategy

This narrative review was developed through a targeted literature search of PubMed, Scopus, and Web of Science databases. Priority was given to peer-reviewed articles published primarily within the last 10–15 years, although seminal earlier studies were included where historically or conceptually relevant. The literature selection focused on studies addressing evolutionary genomics, host–microbe interactions, microbial ecological adaptation, pathobiont behavior, horizontal gene transfer, genome specialization, and comparative aspects of endosymbiosis in relation to the human gut microbiome. Both experimental and review-based studies were considered to provide conceptual integration across microbial ecology, evolutionary biology, and host-associated adaptation. Studies were selected based on their relevance to the central objective of the review, namely understanding how genomic plasticity and ecological pressures shape microbial behavior across the mutualism–pathogenicity spectrum.

## 3. Genomic Adaptations Across Mutualistic, Pathobiont, and Host-Specialized Lifestyles

### 3.1. Mutualists: Genomic Foundations of Cooperation

*Bacteroides thetaiotaomicron* is a model mutualist with a large and highly flexible genome. It harbors over 80 PULs that allow breakdown of diverse dietary and host-derived glycans. These PULs are tightly regulated by hybrid two-component systems, enabling nutrient-sensitive expression and fine-tuned resource use within the gut [23,24]. Functional genetics studies reveal roles of specific glycoside hydrolases in metabolizing pectins, rhamnogalacturonan, and other complex carbohydrates, as well as systems for bile salt resistance that aid in environmental adaptation [9,25,26,27].

*Bifidobacterium longum* subsp. *infantis* specializes in neonatal mutualism through its genomic repertoire of human milk oligosaccharide (HMO) utilization clusters. These gene clusters enable efficient metabolism of HMOs, supporting early colonization and shaping the infant microbiota for immune and gut development [28,29,30]. Unlike reduced obligate endosymbionts, *B. infantis* retains accessory genes for metabolic flexibility, providing resilience during early-life ecological shifts [31].

*Lactobacillus* and *Bifidobacterium* species often encode bile salt hydrolase (BSH) genes and vitamin biosynthesis operons. These contribute to bile detoxification, lipid metabolism, and host micronutrient supply, representing direct host benefits reinforced through genomic adaptation (e.g., B vitamins) [32,33,34] (Table 1).

### 3.2. Pathobionts: Conditional Pathogenicity

Pathobionts such as *E. coli*, *Enterococcus faecalis*, and *K. pneumoniae* exemplify bacteria with dual genomic traits—they possess metabolic versatility alongside latent pathogenic potential [35,36] (Table 1). For instance, some *E. coli* strains encode adhesins, siderophore systems, and urease operons that may remain unexpressed until ecological triggers—like inflammation or dysbiosis—activate pathogenic pathways [35,37].

These organisms often harbor mobile genetic elements—plasmids, prophages, or genomic islands—that encode toxins, antibiotic resistance genes, and secretion systems. In *Enterococcus*, the gelatinase (gelE) and cytolysin operons drive invasiveness [38,39,40]; in *K. pneumoniae*, accessory genomes facilitate capsule production and drug resistance [21,41]. Retained metabolic pathways alongside these virulence modules enable survival across a spectrum of host states.

In addition to gene acquisition and mobile genetic elements, reversible regulatory mechanisms also contribute substantially to ecological plasticity in pathobionts. Phase variation and epigenetic regulation enable bacteria to dynamically alter the expression of surface structures, capsular polysaccharides, adhesins, and virulence-associated factors in response to changing host environments [42,43,44]. Bacteroides fragilis provides a representative example, as phase-variable capsular polysaccharide expression influences immune modulation, colonization efficiency, and persistence within the gut ecosystem [45,46]. Under homeostatic conditions, these regulatory systems may support commensal persistence and immune tolerance, whereas inflammatory or dysbiotic conditions can favor expression of virulence-associated traits such as fragilysin production and epithelial barrier disruption [46,47]. Such reversible regulatory adaptation highlights how conditional pathogenicity can emerge without permanent genomic transformation, reinforcing the concept that ecological plasticity in gut bacteria depends not only on genomic content but also on context-dependent control of gene expression.

### 3.3. Host Specialization and Pathogenic Adaptation Along the Continuum

**Figure 1:**
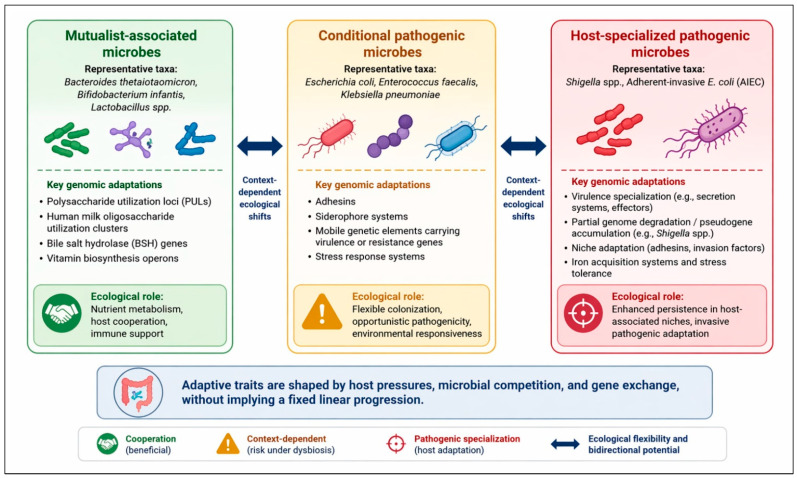
Spectrum of host-associated microbial lifestyles and representative genomic adaptations in gut bacteria. Mutualist-associated, conditionally pathogenic, and host-specialized pathogenic microbes exhibit distinct genomic adaptations that promote persistence under different ecological contexts. The bidirectional relationships indicate ecological flexibility and context-dependent shifts in microbial behavior, rather than a universal linear evolutionary progression toward pathogenicity. Created with BioRender.com.

Among host-adapted pathogenic lineages, some bacteria exhibit genomic features associated with niche specialization and enhanced pathogenicity. These features may include metabolic streamlining, retention of virulence-associated loci, and enhanced dependence on host-derived nutrients. However, unlike obligate parasitic or intracellular symbionts, these organisms generally retain substantial genomic flexibility, and their evolutionary trajectories are better understood as niche specialization rather than irreversible progression toward parasitism [48,49] (Figure 1).

A well-characterized example is *Shigella* spp., which evolved from ancestral *E. coli* lineages and underwent convergent genome degradation marked by pseudogene accumulation, deletion of metabolic functions, and retention of virulence determinants required for epithelial invasion and intracellular survival [50,51]. This genomic streamlining reflects adaptation to a host-restricted pathogenic niche, although it differs fundamentally from the extreme reductive evolution observed in obligate endosymbionts.

Other gut-associated pathobionts demonstrate host-adaptive specialization through acquisition or regulation of virulence determinants rather than through extensive genome reduction. For instance, adherent-invasive *E. coli* (AIEC) strains possess adhesins, iron acquisition systems, and stress-response mechanisms that enhance persistence within inflamed intestinal environments [52,53]. These traits support ecological adaptation to inflammatory niches but do not necessarily indicate reductive evolution toward obligate parasitism.

Similarly, virulence-associated traits in *K. pneumoniae*, including hypervirulence plasmids and enhanced capsule production, can increase pathogenic potential while maintaining the capacity for asymptomatic colonization [54,55,56]. These examples illustrate that host specialization in gut bacteria often involves modulation of accessory virulence traits and ecological adaptation rather than directional evolution toward a fully parasitic lifestyle. Together, these observations indicate that gut microbes can acquire diverse adaptive traits that promote persistence under specific ecological conditions. These adaptations reflect dynamic ecological specialization shaped by host pressures, microbial competition, and gene exchange rather than fixed progression toward parasitism (Table 1).

Host-adapted pathogens also evolve mechanisms that modify host physiology in ways that favor bacterial persistence. For example, *Shigella flexneri* employs specialized secretion systems to manipulate host cytoskeletal pathways and immune responses, facilitating epithelial invasion [57,58]. Likewise, toxigenic *Clostridioides difficile* disrupts epithelial integrity and induces inflammation through TcdA and TcdB toxins, generating conditions that promote bacterial expansion [59,60,61]. Similarly, pks^+^ *E. coli* strains produce colibactin, a genotoxin associated with epithelial damage and altered mucosal homeostasis [62,63,64]. These examples demonstrate how virulence-associated traits can enhance bacterial fitness within the host by exploiting inflammatory or damaged environments, thereby favoring pathogenic specialization under particular ecological conditions.

Together, these observations indicate that gut microbes can acquire adaptive traits that enhance persistence under specific ecological conditions. These adaptations reflect dynamic ecological specialization shaped by host pressures, microbial competition, and gene exchange rather than fixed progression toward parasitism. Although these patterns reveal important forms of host adaptation, they should not be interpreted as evidence of a universal linear evolutionary trajectory from mutualism to parasitism.

## 4. Evolutionary Forces Driving Transitions

**Figure 2:**
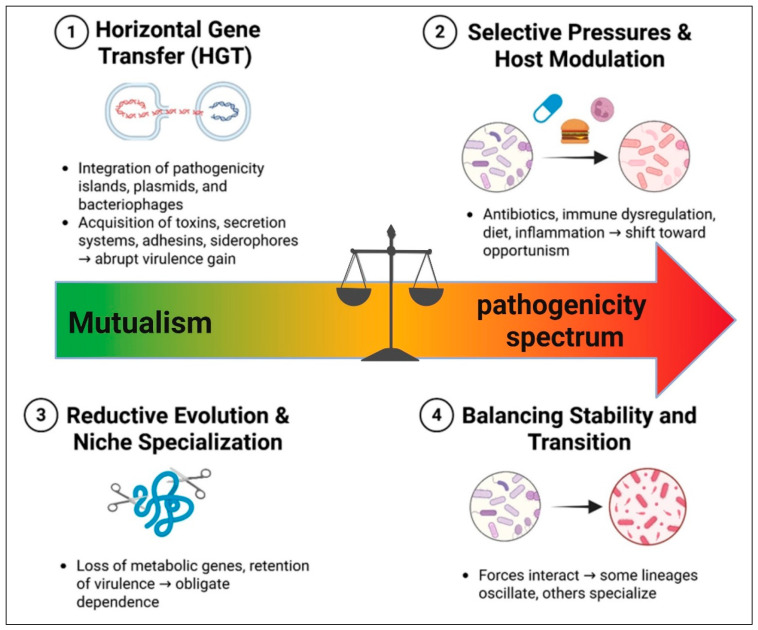
Evolutionary forces driving microbial transitions along the mutualism–pathogenicity spectrum. (1) Horizontal gene transfer (HGT): integration of pathogenicity islands, plasmids, and bacteriophages enables rapid acquisition of toxins, adhesins, secretion systems, and siderophores, resulting in abrupt virulence gain. (2) Selective pressures and host modulation: factors such as antibiotics, immune dysregulation, diet, and inflammation act as environmental filters that shift mutualists toward opportunism. (3) Reductive evolution and niche specialization: the loss of metabolic pathways combined with the retention of virulence determinants reinforces obligate dependence on the host. (4) Balancing stability and transition: these forces rarely act in isolation; instead, their interplay shapes microbial trajectories, with some lineages oscillating between cooperation and pathogenicity while others specialize in parasitism. Created with BioRender.com.

The genomic architecture of host-associated microbes is dynamic, continuously reshaped by evolutionary forces that influence whether lineages maintain cooperative interactions, acquire opportunistic traits, or develop specialized pathogenic adaptations. Rather than following a fixed evolutionary path, microbial genomes reflect adaptive responses to host and environmental pressures that can alter ecological behavior and host outcomes. Exploring these forces provides insight into how microbial traits are gained, lost, or regulated in ways that influence microbial roles within the host ecosystem (Figure 2).

### 4.1. Horizontal Gene Transfer (HGT) and the Acquisition of Virulence Traits

HGT represents one of the most powerful accelerators of microbial evolution, permitting the rapid acquisition of new functions without the need for gradual mutation. In *E. coli*, integration of pathogenicity islands, plasmids, and bacteriophages has repeatedly transformed benign commensals into highly virulent extraintestinal pathogens. These elements often encode toxins, type III secretion systems, adhesins, or siderophores, conferring traits that promote immune evasion, host colonization, and nutrient competition. Similar dynamics are observed in gut-associated *E. faecalis* and *K. pneumoniae*, where mobile genetic elements expand antibiotic resistance and virulence potential. HGT thus acts as a rapid mechanism for acquiring adaptive traits that can alter microbial behavior across the mutualism–pathogenicity spectrum [65,66].

In addition to HGT and genome reduction, ecological adaptation can also emerge through mutations affecting global regulatory systems that rewire existing transcriptional networks. Alterations in transcriptional regulators, two-component signaling systems, quorum-sensing pathways, or stress-response regulators can produce substantial phenotypic shifts without requiring acquisition of new genetic material [67,68,69]. Such regulatory rewiring may alter expression of virulence factors, metabolic pathways, biofilm formation, or immune-evasion mechanisms, thereby enabling rapid adaptation to inflammatory, nutrient-limited, or antibiotic-exposed environments [70,71,72,73,74]. These changes highlight that ecological transitions in gut bacteria may result not only from genomic expansion through HGT, but also from small genetic modifications that reshape the regulation of pre-existing functional pathways.

### 4.2. Selective Pressures and Host-Mediated Modulation

The host environment exerts persistent selective forces that shape microbial plasticity [75,76]. Environmental variables—including diet composition, immune activation, inflammation, antibiotic exposure, and microbial competition—function as ecological “filters” that determine which microbial traits and genetic variants are maintained within the gut ecosystem [77,78,79].

Within-host evolution also contributes substantially to microbial ecological plasticity, particularly under conditions of chronic inflammation, immune dysregulation, or prolonged antibiotic exposure. In these environments, bacterial populations may accumulate adaptive mutations over relatively short evolutionary timescales, promoting persistence within the host [46,80,81]. Of particular importance are hypermutator lineages arising from defects in DNA mismatch repair systems such as *mutS* or *mutL*. These mutations elevate genomic mutation rates and accelerate adaptive diversification, enabling rapid evolution of antibiotic resistance, stress tolerance, metabolic flexibility, and immune-evasion traits [82,83,84,85]. Hypermutator *E. coli* lineages have been observed in chronically inflamed intestinal environments, where increased mutational supply may enhance survival under strong selective pressure despite the potential accumulation of deleterious mutations [86,87]. These observations further illustrate that ecological transitions in gut bacteria may emerge not only through HGT or large genomic rearrangements, but also through rapid within-host evolutionary dynamics driven by mutation-rate variation.

### 4.3. Reductive Evolution and Niche Specialization

In contrast to gene acquisition, reductive evolution streamlines genomes by eliminating unnecessary functions in a protected host environment. This process can promote host specialization by reducing unnecessary functions in stable host-associated environments, sometimes increasing dependence on host-derived resources [88,89]. *Shigella* spp. provides an example of partial genome degradation associated with adaptation to a host-restricted pathogenic niche, including pseudogene accumulation and loss of metabolic flexibility while retaining virulence determinants required for invasion [51].

### 4.4. Balancing Stability and Transition

These forces rarely operate in isolation. Newly acquired virulence determinants through HGT may undergo host-driven amplification, while prolonged adaptation can culminate in genome reduction and long-term specialization. Importantly, microbial populations within the same species may diverge, with some lineages oscillating between mutualism and pathogenicity, while others undergo increasing host specialization and pathogenic adaptation. This interplay explains both the remarkable versatility of many gut commensals and the persistence of host-restricted pathogens. Understanding these intertwined mechanisms underscores the evolutionary mechanisms that shape microbial adaptation and influence host–microbe interactions [90].

Adaptive evolution within the gut microbiome may proceed through multiple selective dynamics rather than through single deterministic evolutionary events. In some cases, ecological pressures such as antibiotic exposure produce hard selective sweeps, in which a single advantageous lineage carrying a resistance determinant rapidly expands and displaces competing populations, thereby reducing microbial diversity [91,92,93,94]. In contrast, soft selective sweeps occur when multiple pre-existing adaptive variants or independently acquired resistance determinants simultaneously increase in frequency within the microbial community, preserving greater genetic heterogeneity [95,96,97]. Beyond single-gene adaptation, gut bacteria may also undergo polygenic adaptation, where numerous small-effect mutations across regulatory, metabolic, stress-response, and membrane-associated pathways collectively improve fitness under inflammatory or nutrient-altered conditions [46,90,98,99]. For example, adaptation to repeated antibiotic exposure may involve not only acquisition of resistance genes through HGT, but also incremental modifications in transcriptional regulation, efflux activity, metabolic efficiency, and biofilm formation [100,101,102]. These evolutionary dynamics illustrate that ecological adaptation in the gut ecosystem often emerges through diverse population-level processes operating simultaneously across microbial communities.

## 5. Ecological and Clinical Implications of Context-Dependent Microbial Adaptation

Evolutionary shifts in gut microbes, whether driven by HGT, genome reduction, or adaptive mutations, carry profound ecological and clinical consequences that extend far beyond the molecular events themselves. Within the gut ecosystem, these shifts frequently alter the balance between symbiosis and pathogenicity, reshaping the relationships that commensal organisms maintain with their host.

*Bacteroides fragilis* provides a representative example of how gut bacteria can transition along the mutualism–pathogenicity spectrum in response to ecological context. Under homeostatic conditions, non-toxigenic strains expressing polysaccharide A (PSA) contribute to immune regulation and intestinal tolerance through modulation of host T-cell responses and anti-inflammatory signaling pathways [103,104,105,106]. In contrast, enterotoxigenic strains harboring the bft pathogenicity island produce fragilysin, a metalloprotease that disrupts epithelial barrier integrity, activates inflammatory signaling pathways, and contributes to colitis and colorectal carcinogenesis under dysbiotic conditions [107,108,109,110]. Importantly, these contrasting behaviors are not determined solely by species identity, but instead reflect strain-level genomic variation, regulatory adaptation, and host environmental conditions. Inflammatory stress, immune dysregulation, antibiotic exposure, and altered microbial competition may favor expression of virulence-associated traits, illustrating how ecological pressures can shift the functional role of the same bacterial species from mutualistic colonizer to pathogenic pathobiont.

Similarly, certain *E. coli* lineages, once considered benign members of the gut microbiome, have acquired the *pks* genomic island encoding colibactin, a genotoxin capable of inducing DNA damage and accelerating colorectal cancer development [62,111,112]. These transformations illustrate how evolutionary innovations can convert ordinary commensals into “pathobionts,” organisms that exist on the threshold between mutualism and pathogenicity and whose ecological impact depends heavily on host context.

Environmental pressures such as antibiotics, dietary habits, and immune status can reshape microbial communities, often tipping the balance between harmless coexistence and harmful disease. These shifts demonstrate how evolutionary forces operate in real-world settings, with direct consequences for both health and disease [113].

Antibiotic exposure provides a clear example of how external pressures can accelerate microbial transitions. Broad-spectrum antibiotics not only eliminate pathogenic bacteria but also disrupt the protective commensals that maintain ecological balance. The loss of beneficial *Clostridia* or *Bifidobacterium* creates ecological gaps that can be rapidly filled by opportunistic species. One well-known outcome is the overgrowth of *C. difficile*, a bacterium that takes advantage of this disturbed ecosystem to cause life-threatening colitis. Similarly, antibiotic pressure has fueled the rise in multidrug-resistant *Enterobacteriaceae*, where survival depends not only on resistance genes but also on the ability to exploit disturbed host niches [114,115,116].

Dietary changes also play a central role in shaping microbial behavior. A diet rich in fiber supports a beneficial microbiome that produces short-chain fatty acids (SCFAs), which help protect the gut lining and regulate immunity [117,118]. By contrast, Western-style diets, high in fat and low in fiber, reduce these protective populations and allow potentially harmful microbes to expand. For example, blooms of *E. coli* and other pathobionts have been linked to inflammatory bowel disease (IBD) and metabolic syndrome in individuals consuming low-fiber diets [119,120,121,122,123]. These findings show how food choices can influence microbial ecology in ways that promote or prevent disease.

Importantly, many of these ecological shifts are accompanied by functional metabolic changes that provide a mechanistic link between microbial genomic adaptation and host disease phenotypes. Genomic variation affecting carbohydrate metabolism, stress-response systems, regulatory networks, or horizontally acquired metabolic pathways can alter the production of bioactive metabolites such as SCFAs and secondary bile acids. For instance, reductions in butyrate-producing populations may impair epithelial barrier integrity and immune regulation, thereby increasing susceptibility to inflammatory disorders. Likewise, adaptive changes in microbial bile acid transformation pathways can influence host metabolic and inflammatory signaling through FXR- and TGR5-associated mechanisms. These observations illustrate how microbial evolutionary adaptation extends beyond genomic change alone, shaping host physiology through altered ecological interactions and metabolic outputs.

The immune system acts as another key driver in microbial evolution. In healthy individuals, immune defenses keep many microbes in check, preserving a balance between tolerance and control. However, in conditions of immune suppression—such as HIV infection, cancer chemotherapy, or immunosuppressive drugs—commensals can shift toward pathogenicity. Organisms like *Candida albicans*, *K. pneumoniae*, and *E. faecalis* often live harmlessly in healthy hosts but can cause severe bloodstream infections, pneumonia, or sepsis when the immune system is weakened [124,125,126,127]. These clinical cases illustrate how the same microbe can alternate between symbiosis and parasitism depending on the host context.

Therapeutically, the microbiome’s evolutionary flexibility poses both obstacles and advantages. Rapid acquisition of resistance undermines traditional antibiotics, while unpredictable pathobiont activation in vulnerable patients complicates treatment strategies. On the upside, insights into microbial evolution are driving precision interventions. Fecal microbiota transplantation (FMT), for instance, restores community balance and is widely used for recurrent *C. difficile* infection—with evidence for lasting microbiome recovery and potential broader applications [128,129]. Similarly, banked phages or bacteriophage-enriched transplants show promise in reshaping microbial networks, particularly in reprogramming harmful populations [130,131].

In many cases, distinguishing whether pathobiont expansion is a primary driver of disease or a secondary consequence of ecological disruption remains challenging, particularly in human association studies where causality is difficult to establish.

In sum, microbial behavior in the gut is strongly influenced by ecological dynamics shaped by diet, immunity, antibiotic exposure, and microbe–microbe interactions. These factors can alter the balance between beneficial and pathogenic outcomes by changing community structure and microbial activity.

## 6. Lessons from Classical Endosymbionts: Parallels and Contrasts in Human Gut Bacteria

Endosymbiotic bacteria like *Buchnera* in aphids and *Wolbachia* in insects offer powerful models to understand long-term symbiosis. Examining their evolutionary genomics provides valuable context for interpreting human gut microbial dynamics.

### 6.1. Genome Reduction and Functional Specialization

One hallmark of classical insect endosymbionts such as *Buchnera aphidicola* is extensive genome reduction associated with long-term intracellular residence and obligate host dependence. These organisms have lost large numbers of genes involved in autonomous metabolism, environmental sensing, and DNA repair, while retaining functions essential for host benefit, such as amino acid biosynthesis [4,132,133]. This process reflects an advanced stage of metabolic integration between symbiont and host.

In human gut bacteria, comparable patterns of specialization may occur, but they are generally less extreme and arise under markedly different ecological conditions. Certain pathogenic lineages, such as *Shigella* spp., exhibit genome degradation relative to ancestral *E. coli*, characterized by pseudogene accumulation and loss of metabolic functions associated with environmental versatility [50,51]. However, unlike insect endosymbionts, this streamlining supports pathogenic specialization rather than obligate mutualistic dependence.

Thus, while both endosymbionts and host-adapted gut bacteria may undergo functional specialization, the evolutionary outcomes differ substantially. Insect endosymbionts evolve toward stable metabolic interdependence with the host, whereas gut bacteria more commonly retain genomic plasticity that permits shifts between commensalism and pathogenicity [134,135,136].

Although genome degradation in pathogens such as *Shigella* spp. is frequently interpreted as evidence of host specialization, whether these changes represent directional evolutionary progression or context-dependent adaptation remains unresolved.

### 6.2. Mutation, Drift, and Genetic Isolation

Insect endosymbionts typically experience strong genetic drift due to small effective population sizes and frequent bottlenecks during vertical transmission. This leads to accumulation of deleterious mutations, AT-biased mutational spectra, and continued gene loss—a “degenerative” path known as Muller’s ratchet [132,133]. Endosymbiont genomes also show reduced recombination and suppressed HGT, further limiting adaptive flexibility [132].

In contrast, human gut microbiota often inhabits large and dynamic populations, with frequent opportunities for recombination and horizontal gene flow—especially via mobile genetic elements, phages, and plasmids. Pathobionts exploit this genomic plasticity to acquire antibiotic resistance and virulence traits [134,135,136]. Meanwhile, true mutualists in the gut, such as *Bacteroides* or *Bifidobacterium*, maintain more stable genomes but still benefit from gene exchange, unlike the genetic isolation seen in insect endosymbionts [137,138,139,140].

### 6.3. Horizontal Gene Transfer Versus Static Lineages

Unlike *Buchnera*, which lacks mobile elements and recombination machinery, *Wolbachia* genomes can be surprisingly plastic. Some strains of *Wolbachia*, including the bedbug-associated wCle, have acquired vitamin-biosynthesis operons (e.g., biotin). This operon, likely obtained via lateral transfer from coinfecting bacteria like *Cardinium* or *Rickettsia*, enabled *wCle* to provision its host with an essential vitamin, facilitating a shift toward obligate mutualism [141,142,143,144]. Moreover, *Wolbachia* DNA frequently inserts into host genomes, and bacteriophage WO mediates lateral gene transfer among *Wolbachia* strains [142]. Genomic analyses further reveal that similar operons are present in planthopper-associated *Wolbachia*, conserving their capacity to provide biotin and riboflavin to hosts [6,143]. These findings underscore how, in *Wolbachia*, HGT can enable the emergence of beneficial symbiotic functions rather than the opportunistic acquisition of pathogenic traits.

Human gut microbiota likewise showcases high levels of HGT, with mobile resistance islands and virulence factors disseminating between taxa [136,145]. These transfers can drive swift adaptive responses to environmental or clinical pressures, highlighting the gut as a dynamic arena for microbial evolution.

Thus, while HGT is a common evolutionary mechanism in both contexts, the functional consequences diverge sharply. In human gut microbiota, HGT tends to be harnessed for rapid adaptation to changing environments—often featuring antimicrobial resistance or pathogenicity. In *Wolbachia*, however, the acquisition of nutritional biosynthesis pathways via HGT supports cooperative host–symbiont relationships, fostering mutualism rather than virulence.

### 6.4. Comparative Insights

Comparisons between classical insect endosymbionts and human gut bacteria provide useful conceptual insights into how microbes adapt to long-term host association, but the parallels should not be interpreted as equivalent evolutionary trajectories. In both systems, host association can favor specialization, retention of host-relevant traits, and loss of functions that are no longer advantageous in the host environment [132,133,146].

However, the extent and consequences of these processes differ substantially. Insect endosymbionts typically undergo irreversible genome reduction driven by vertical transmission, small effective population sizes, and strong host dependence [132,133]. By contrast, human gut bacteria generally exist in large, dynamic populations where HGT and recombination maintain substantial genomic plasticity [134,135,136]. This flexibility allows gut microbes to alternate between mutualistic and pathogenic behaviors in response to ecological pressures rather than becoming locked into obligate dependence.

DNA repair systems further illustrate these contrasting evolutionary trajectories. In highly specialized intracellular symbionts such as *Buchnera aphidicola*, reductive genome evolution is frequently accompanied by progressive loss of DNA repair and recombination pathways, contributing to reduced genomic plasticity and long-term evolutionary stability within a constrained host-associated niche [133,147,148]. In contrast, gut-associated pathobionts exposed to chronic inflammation, oxidative stress, or prolonged antibiotic exposure may experience selective pressures favoring transient hypermutability through defects in mismatch repair systems such as *mutS* or *mutL*. Rather than promoting genomic stabilization, these mutator phenotypes can accelerate adaptive diversification, facilitating rapid evolution of antibiotic resistance, metabolic flexibility, stress tolerance, and immune-evasion mechanisms within fluctuating gut environments [149,150,151]. Thus, although both systems involve alterations in DNA repair capacity, the evolutionary outcomes differ fundamentally: obligate endosymbionts undergo reductive stabilization associated with ecological specialization, whereas gut bacteria often retain or exploit mutational flexibility to preserve adaptability in dynamic host ecosystems.

Therefore, the value of the endosymbiont comparison lies primarily in illustrating general principles of host adaptation and specialization, rather than implying that gut microbes follow the same deterministic path toward obligate symbiosis or parasitism.

A comparative summary of evolutionary features in classical insect endosymbionts and human gut bacteria is presented in Table 2.

## 7. Conclusions and Future Perspectives

Evolutionary genomics highlights the remarkable adaptability of human gut bacteria across a spectrum of host-associated lifestyles, ranging from beneficial mutualists to opportunistic pathogens. While some lineages exhibit host-specialized traits such as metabolic streamlining, virulence acquisition, or niche adaptation, these changes do not necessarily represent a linear evolutionary progression toward parasitism. Instead, they reflect flexible and context-dependent responses to ecological pressures within the gut environment.

Comparisons with classical insect endosymbionts offer a useful conceptual framework for understanding host-associated specialization, but the evolutionary dynamics of human gut bacteria are fundamentally distinct. Unlike obligate endosymbionts, gut microbes generally retain substantial genomic plasticity that enables reversible shifts in ecological behavior depending on host and environmental conditions.

By integrating evidence from comparative genomics, microbial ecology, and host–microbe interaction studies, this review highlights that transitions along the mutualism–pathogenicity spectrum emerge through interconnected evolutionary and ecological processes rather than deterministic trajectories. Horizontal gene transfer, regulatory adaptation, microbial competition, host-mediated selection, and environmental perturbations collectively shape microbial persistence, specialization, and context-dependent behavioral shifts within the gut ecosystem.

Recognizing this ecological flexibility is essential for understanding how commensal organisms contribute to health in one context yet promote disease in another. Future research integrating longitudinal microbiome profiling, strain-resolved genomics, functional multi-omics, and experimental ecological modeling will be important for clarifying how microbial adaptation influences disease susceptibility, therapeutic response, and long-term host–microbe stability.

From a translational perspective, improved understanding of microbial evolutionary dynamics may support the development of microbiome-informed therapeutic strategies, including precision dietary interventions, targeted microbiota modulation, bacteriophage-based approaches, and personalized medicine frameworks aimed at preserving microbial homeostasis while limiting pathobiont expansion. More broadly, incorporating evolutionary and ecological principles into microbiome research may improve future therapeutic design by accounting for the adaptive flexibility and resilience of host-associated microbial communities.

## Figures and Tables

**Table 1:** Representative genomic adaptations associated with different host-associated microbial lifestyles in the human gut.

Lifestyle Category	Representative Taxa	Genomic Adaptations	Ecological Implication
Mutualist-associated	*B. thetaiotaomicron*	PULs, glycoside hydrolases	Host–microbe cooperation
	*B. infantis*	HMO utilization clusters	Infant gut colonization
Conditional pathogenic	*E. coli*	Adhesins, siderophores	Opportunistic activation
	*E. faecalis*	Cytolysin, gelE	Opportunistic pathogenicity
Host-specialized pathogenic	*Shigella* spp.	Genome degradation, invasion loci	Invasive pathogenic adaptation
	AIEC	Adhesins, iron acquisition systems	Inflammation-associated persistence
	*K. pneumoniae*	Hypervirulence plasmids	Increased pathogenic potential

**Table 2:** Comparative host-adaptation patterns in insect endosymbionts and human gut microbes.

Host-Adaptation Principle	Insect Endosymbionts	Human Gut Microbes	Key Distinction
Genome specialization	Extensive reductive evolution with retention of host-beneficial genes	Variable specialization with limited genome degradation in some lineages	Gut microbes retain greater genomic plasticity
Host association	Obligate long-term intracellular dependence	Dynamic colonization influenced by ecological context	Most gut microbes are facultatively associated
Functional adaptation	Metabolic complementation to host	Colonization, immune modulation, virulence adaptation	Outcomes range from mutualism to pathogenicity
Transmission mode	Primarily vertical	Primarily horizontal	Horizontal transfer maintains adaptability
Evolutionary flexibility	Low, often irreversible	High, reversible	Gut microbes can shift ecological roles
DNA repair and mutational dynamics	Progressive loss of DNA repair and recombination systems	Stress-associated hypermutability and adaptive regulatory evolution	Endosymbionts favor genomic stabilization, whereas gut microbes may exploit adaptive mutational flexibility

## Data Availability

No new data were created or analyzed in this study. Data sharing is not applicable to this article.
